# Supplementary material for: Predictive role of the peripheral blood inflammation indices neutrophil-to-lymphocyte ratio (NLR), platelet-to-lymphocyte ratio (PLR), and systemic immunoinflammatory index (SII) for age-related cataract risk
Source: PLoS One. 2024 Nov 18;19(11):e0313503. doi: 10.1371/journal.pone.0313503 (PMC11573120; doi:10.1371/journal.pone.0313503)
Supplement: Attachment — Submitted filename: review report PONE.docx [file pone.0313503.s001.docx]

Dear Editor and Authors,

In this manuscript, the authors compared the NLR, SII, and PLR levels of 935 cataract patients registered in the NHANES database with a population of 8887 participants.

1. The purpose of the study is not clear. Why did the authors investigate NLR, SII, and PLR in cataract patients in this study?

2. Did the cataract group consist of those who had undergone cataract surgery, or were both those who had undergone cataract surgery and those who had not? If the participants had already undergone cataract surgery, what was the purpose of assessing NLR, SII, and PLR in these individuals, and what would this do?

3. How do the authors explain the lower risk of cataract in those with BMI <30?

4. The authors should provide insight into how NLR and SII can be used in clinical practice for cataract. Should everyone with elevated levels of these parameters be screened for cataract? If so, these parameters will increase in numerous chronic and acute inflammatory conditions and are not specific to cataracts. The authors consider how to overcome this handicap. In addition, older age is more associated with cataract risk than these parameters, and if they are to be used for cataract screening, it seems more cost-effective to screen the elderly population. If they are recommended for diagnosis, it is impossible to diagnose cataract by evaluating these parameters. Because increases in these parameters can be detected in many people due to many reasons.

5. Ratios such as NLR and SII are calculated from data obtained from complete blood counts and are continuously variable values that are affected by many conditions and very quickly. As the authors also mentioned, examining these parameters only once in a chronic pathological condition such as cataract is devoid of establishing a cause-effect relationship.

6. The English language contains many grammatical and typo errors.
